# The Adelaide Hospital and the British Medical Association

**Published:** 1897-07-10

**Authors:** 


### THE ADELAIDE HOSPITAL AND THE BRITISH MEDICAL ASSOCIATION.

We hear from Adelaide that the South Australian Government has instructed a firm of solicitors in London to take legal steps to prevent the British Medical Association expelling Dr. Ramsay Smith and Dr. Leith Napier, who, in accepting office at the Adelaide Hospital, are stated to have "acted in opposition to the strongly-expressed feeling and opinion of the medical profession in Adelaide." We do not think, however, that it will be necessary for these legal gentlemen to take any action, for we cannot believe that the British Medical Association will be so ill-advised as to attempt the exercise of such disciplinary powers as the power of expulsion may possible give it, in reference to such a much-disputed question as that of the Adelaide Hospital squabble.

In regard to the propriety of the new staff accepting the appointments offered to them by the Hospital Board, a body directly nominated by the South Australian Government, it may be well to recall to mind the position of affairs at the time that the appointments were made. There can be no doubt that the members of the new staff, in accepting their appointments, went in opposition to the expressed wish of the great majority of the medical men practising in Adelaide; but unless we are prepared to admit that no one ought to act contrary to the expressed wish of the majority—an admission which, if generally acted on, would tend to fossilise all medical practice—we must allow to each man the right to consider both the nature of the question and the grounds on which the majority have arrived at their conclusions. If this is done in regard to the Adelaide Hospital dispute, we find that the origin of the quarrel was not any important ethical question but a mere detail of hospital management, in regard to which it was perfectly open to everyone to hold his own opinion. The case is clearly put in a letter by Mr. Victor Horsley which appeared in the *British Medical Journal* on May 30th, 1896. In it he describes how a difficulty arose in regard to the promotion of a certain nurse, and matters ultimately reached such a pitch that a Royal Commission was appointed to inquire into them; how this Commission insisted on the reinstatement of two nurses who had been suspended; and how, on the Board refusing to carry out the instructions of the Government, the members of the old Board were not reappointed and the nurses they had suspended were reinstated. So far the medical staff did not appear to have intervened. But on the new Board of Management no representative of the staff was appointed, while among its members "was a practitioner whose name had been removed from the list of members of the local branch of the British Medical Association. This appointment aroused the indignation of the staff, who thereupon resigned in a body." Here then we have the reason which led to the resignation of the staff, viz., the omission from the Board of Management of any member of the medical staff, and the appointment on that Board of a person who was distasteful to the staff. We venture to suggest that it is still quite an open question whether it is a good plan to have mixed boards, and as for a medical staff dictating to the governors of a hospital as to what outside persons should be placed on the Board of Management, such a thing is utterly unheard of in London. In any case these are not reasons which could give to any body of practitioners, however unanimous they might be, the right to dictate to anyone who differed from them; and it must be remembered that, whatever other facts may have been known in Adelaide, this represented what was known in England at the time that these "black sheep" accepted their appointments.

On October 3rd, 1896, we published an article on the Adelaide Hospital and the Government of South Australia, written by a contributor who from his position in the British Medical Association appeared to us likely to know all that was going on. Almost simultaneously an article in the same strain, and apparently from the same source, appeared in the *British Medical Journal*. The object of both these articles was to illustrate the evils which arise from Government interference in hospital management, but the history told in both of them showed how thoroughly political the affair had been from the beginning, and that the action against which the medical staff protested was one taken, however wrongly, by the responsible Government of the country after the matter had been investigated and reported on by a Royal Commission. Unless, then, there is something in the background which has not appeared, any attempt to expel men from the Association because they have accepted Government appointments offered to them under such circumstances would seem a very high-handed proceeding. The British Medical Association has better and more appropriate work to do than fighting the labour party in South Australia. The whole question is a very hot potato, which had better be dropped before the Association burns its fingers.

